# Profiles of Asian American parent‐ and adolescent‐reported ethnic‐racial socialization: A person‐centered analysis

**DOI:** 10.1111/jora.70176

**Published:** 2026-04-07

**Authors:** Pearl Sun, Cixin Wang, Charissa S. L. Cheah, Ashlyn Michot, Louisa Wetzel

**Affiliations:** ^1^ Department of Counseling Higher Education, and Special Education University of Maryland College Park Maryland USA; ^2^ Department of Psychology University of Maryland Baltimore County Baltimore Maryland USA; ^3^ Department of Psychology University of Maryland College Park Maryland USA

**Keywords:** Asian American families, ethnic–racial socialization, parent–child dyad

## Abstract

Ethnic–racial socialization (ERS), the communication of parent worldviews about race and ethnicity to children, is important for youth development. Most research only examines parent or youth reports of these socialization practices in isolation, and few have focused on Asian American families. The present study addresses these gaps, incorporating both parent and youth reports of ethnic–racial socialization practices in Asian American families. Participants were 466 Asian American parents (*M*_age_ = 46.52 years, SD = 5.21), including 317 Chinese Americans, 118 Korean Americans, and 31 Filipino Americans, and their adolescents (*M*_age_ = 14.72 years, SD = 1.91). Most parents (94%) were born outside of the United States while most adolescents (80%) were born in the United States. Parents comprised 377 mothers (81%) and 89 fathers (19%). Participants resided across 30 U.S. states. Dyads completed the *Asian American Parental Racial–Ethnic Socialization Scale* (*Cultural Diversity & Ethnic Minority Psychology*, 2016, 22, 417), measuring seven ERS dimensions. We conducted latent profile analyses to identify patterns of parent and child reports of parental ERS messages and examined associations between profile membership and Asian American youth adjustment (mental health, academic grades, social competence, ethnic identity development) using the BCH procedure in Mplus. Model fit indices supported a four‐profile solution. Adolescents in the High Positive/Low Negative ERS and the Moderate Positive/Low Negative ERS profiles displayed significantly lower levels of anxiety and depression symptoms compared to adolescents in the Parent Low Youth Moderate Negative ERS profile, as well as significantly better academic outcomes compared to adolescents in the Parent High Youth Moderate All ERS and the Parent Low Youth Moderate Negative ERS profiles. Adolescents in the High Positive/Low Negative ERS profile exhibited more positive ethnic identity outcomes compared to adolescents in the Parent High Youth Moderate All ERS and the Parent Low Youth Moderate Negative ERS profiles. Furthermore, adolescents in the High Positive/Low Negative ERS profile scored higher on social competence compared to youth in all other profiles. This study provides the first empirical evidence on how both the content (type of ERS message) and process (parent‐adolescent agreement on ERS) of ERS transmission impact Asian American youth adjustment. It is not only important for parents to promote positive ERS practices to support youth, but also to ensure that these practices are effectively perceived by their youth.

## INTRODUCTION

The transmission of cultural messages from parents to children constitutes a fundamental developmental process, particularly for culturally diverse families navigating multiple cultural contexts (García Coll et al., 1996). This transmission often occurs through ethnic–racial socialization (ERS), the explicit and implicit communication of messages about race and ethnicity in families (Hughes et al., 2006), which shapes youth development and adjustment (Green et al., 2022; Park et al., 2021). ERS is a multidimensional construct that can represent positive (e.g., maintenance of heritage culture, cultural pluralism, promotion of equality) and negative (e.g., avoidance of outgroups, minimization of race) practices, distinguished by their associations with youth outcomes (Juang et al., 2016).

The present investigation was guided by the integrative model of developmental competencies in minority youth (García Coll et al., 1996) and the Navigating Marginalization and Invisibility as Asian Americans theory (Mistry & Kiyama, 2021). The integrative model places social positionality at the center, rather than the periphery, of minoritized children's development, and frames social variables (e.g., race/ethnicity, social class, ERS) as critical in shaping developmental competencies (e.g., ability to cope with experiences of racism; García Coll et al., 1996). Complementing this model, Mistry and Kiyama (2021) represent Asian American youth ethnic–racial identity development through three intersecting domains: features of physical and social environments (acknowledging intragroup heterogeneity and social stratification), master narratives (pervasive stereotypes like “model minority” and “perpetual foreigner”) as meaning making, and developmental processes (ethnic–racial identity formation and navigating marginalization). The model (Mistry & Kiyama, 2021) recognizes the interplay of youth personal development, social context (e.g., stratification), and negotiation through master narratives and creating alternative narratives (e.g., through parental ERS).

While extant literature has examined how ERS may impact youth development, researchers often examine parent or youth reports of ERS practices in isolation. However, parent and youth reports can differ significantly, and parent‐adolescent discrepancies in ERS can reflect variations in how cultural messages are transmitted and received within families (Chen et al., 2021; Green et al., 2022). Discrepancies where adolescents report lower levels of parental ERS than their parents may reflect adolescents misinterpreting or rejecting their parents' ERS, whereas higher levels of adolescent‐reported ERS may reflect adolescents processing and understanding their parents' socialization messages (Chen et al., 2021). While the importance of ERS in culturally diverse families is well documented (Hughes et al., 2006), less research has examined patterns of agreement and disagreement between parent and youth perceptions of ERS practices, particularly within Asian American families. The present study examined the joint influences of different dimensions of ERS and patterns of parent‐adolescent agreement on ERS in shaping Asian American youth development.

### Impact of different ERS strategies on Asian American youth

Anti‐Asian American sentiments, fueled by misinformation regarding the COVID‐19 pandemic, have increased in frequency and severity, leading to detrimental effects on Asian American youth adjustment (Cheah et al., 2020, 2023), such as increased levels of depression and suicidal ideation (Park et al., 2021). Through appropriate ERS practices, parents can shield their Asian American youth from the negative consequences of exposure to discrimination and stereotyping (Cheah et al., 2021). To address the growing need to understand Asian American ERS practices, the Asian American Parental Racial–Ethnic Socialization Scale (Juang et al., 2016) was developed identifying seven domains of ERS that Asian American parents may promote.

In this manuscript, we consider the following ERS strategies theoretically positive based on Juang et al.'s (2016) theory and empirical data: maintenance of heritage culture, cultural pluralism, promotion of equality, and becoming American. Maintenance of heritage culture (preservation of Asian culture) and cultural pluralism (appreciation for diversity) have been linked to positive youth outcomes, such as reduced psychological distress (Atkin et al., 2019), less depression and anxiety (Liu & Lau, 2013), less suicidal ideation (Park et al., 2021), increased social competence (Tran & Lee, 2010), and higher academic performance (Wang, Smith, et al., 2020). Promotion of equality (belief that people are equal and deserve equal treatment) is conceptually positive for child development (Juang et al., 2016). It has been linked to increased self‐worth and academic commitment (Neblett et al., 2009) and has been found to buffer the effects of discrimination on youth depression and self‐esteem (Jiménez & Glover, 2023).

Limited research has examined the link between youth mental health and becoming American, defined as parents' implicit modeling of engagement in American culture (Atkin & Yoo, 2021; Juang et al., 2016). Theoretically, these practices resemble integrative acculturation, which emphasizes the combination of new American values with existing heritage cultural values and is associated with better mental health outcomes (Choy et al., 2020; Nakash et al., 2012). In Juang et al.'s (2016) study with Asian American college students, parents' practice of becoming American was significantly and positively correlated with parents' practice of promotion of equality and cultural pluralism, as well as their child's ethnic identity and pluralistic orientation. As a result, we classified becoming American as a positive ERS practice, although additional research is needed to further evaluate its impact. Regarding the usage of becoming American with maintenance of heritage culture, Juang et al.'s (2016) study with a sample of young adults' (79% U.S.‐born) reports of their parents' (81% immigrants) ERS practices found that parents' practice of becoming American was negatively correlated with their practice of avoidance of outgroups and maintenance of heritage culture. However, in a prior study with Chinese American parents (97% first‐generation immigrants; Zhu et al., 2024), and in our study (94% immigrant parents), becoming American and maintenance of heritage culture were significantly positively correlated. While mixed, these findings suggest that immigrant parents may practice becoming American and maintenance of heritage culture simultaneously.

We also propose the following three ERS strategies are theoretically negative: avoidance of outgroups, minimization of race, and awareness of discrimination. However, it should be noted that some practices (especially awareness of discrimination) had mixed findings. Avoidance of outgroups (encouragement to avoid and distrust other racial–ethnic groups) and minimization of race (endorsement of a racially ‘colorblind’ ideology; Juang et al., 2016) practices have been linked to more mental health difficulties (Atkin et al., 2019; Cheah et al., 2023; Liu & Lau, 2013; Park et al., 2021; Zhu et al., 2024), more social–emotional and behavioral problems (Ren et al., 2022), and decreased social competence (Tran & Lee, 2010). Ethnic–racial minoritized individuals report avoiding majority‐group environments due to fears of discrimination and discomfort (Kraus et al., 2024). Past discriminatory experiences and anticipated bias can cause distress during intergroup interactions (Major et al., 2013), which has been linked to increased symptoms of depression and anxiety (Wang & Narcisse, 2025). It should be noted that avoidance may be protective in certain contexts to prevent real or anticipated unsafe, discriminatory exposures. However, in the long run, avoidance of outgroup messages fails to equip youth with the skills to cope with discrimination, interact with diverse peers and adults, or empower them to develop positive social identities or positive relationships (Cheah et al., 2021). Consequently, when faced with discrimination, youth are more likely to withdraw from individuals who could otherwise serve as valuable support systems, believing that suffering in silence and isolation is their safest option (Atkin et al., 2019; Kiang et al., 2018).

Awareness of discrimination messages (knowledge that others may try to limit their success because of anti‐Asian sentiments) has been linked to mixed findings, such as higher levels of stress for immigrant youth (Saleem et al., 2022), more depressive symptoms (Xie et al., 2021), lower self‐esteem (Hughes et al., 2006), but also better academic performance (Wang, Smith, et al., 2020). However, awareness of discrimination messages that are delivered without accompanying coping strategies and ways to address this anticipated discrimination, as operationalized by the measurement of this construct in the current study (Juang et al., 2016), are more likely to lead to greater anxiety (Ren et al., 2022).

### Factors influencing Asian American parental ERS

Asian American parents' ERS practices emerge from complex sociocultural contexts that shape the messages they communicate to youth. Mothers tend to engage in more frequent ERS conversations than fathers across most domains (Atkin et al., 2023; Kiang et al., 2022), reflecting gendered patterns of child‐rearing responsibilities in Asian American families. Additionally, cultural values of collectivism and family reputation are associated with increased emphasis on awareness of discrimination (Kim et al., 2025), while greater acculturation is associated with more frequent use of promotion of equality messages and less frequent use of minimization of race and avoidance of outgroups messages (Kim et al., 2024; Wong et al., 2024). Internalized racism, which can manifest among Asian Americans through endorsement of model minority stereotypes, has also been linked to increased negative ERS practices (avoidance of outgroups, minimization of race) and decreased positive ERS practices (becoming American, cultural pluralism, promotion of equality; Kim et al., 2024, 2025). The interplay of these complex processes underscores the importance of examining patterns of both parent‐ and youth‐reported ERS, as parents' sociocultural backgrounds shape the messages they choose to convey and subsequently their youth's understanding and alignment with them. While these sociocultural factors provide important context, our goal was not to model these predictors of ERS. Rather, the current study focuses on examining naturally occurring patterns of ERS practices and their associations with youth outcomes without controlling for demographic or sociocultural characteristics.

### Examining ERS through person‐centered approaches

Taken together, the integrative model (García Coll et al., 1996) and the Navigating Marginalization and Invisibility framework (Mistry & Kiyama, 2021) provide a strong theoretical basis for expecting heterogeneity in how ERS unfolds within Asian American families. García Coll et al. (1996) emphasize that developmental processes are shaped by intersecting social positions and contextual demands, suggesting that families vary meaningfully in the types of cultural messages they prioritize and the processes for transmitting these messages. Similarly, Mistry and Kiyama (2021) highlight that Asian American youth and families navigate multiple and sometimes conflicting master narratives within diverse social environments, implying that ERS reflects ongoing family‐level negotiation and meaning making rather than a uniform process. Thus, both frameworks infer the existence of distinct, naturally occurring patterns of ERS characterized by different combinations of ERS message content and levels of parent‐adolescent agreement. Consistent with this expectation, latent profile analysis (LPA) discovers naturally occurring ERS patterns and can capture the varied experiences of adolescents and their families. By identifying profiles that may be uniquely shaped by individual or family‐level factors such as social positioning or ethnic–racial identity formation (García Coll et al., 1996; Mistry & Kiyama, 2021), this approach acknowledges heterogeneity across different families rather than assuming uniform patterns. Moreover, person‐centered approaches offer a more ecologically valid representation of how ERS may be experienced within families: in reality, adolescents do not receive ERS messages in isolation (Atkin et al., 2022). A person‐centered analysis can capture this complexity by examining how different ERS messages may cluster within individuals, revealing meaningful socialization patterns that shape development (Christophe & Stein, 2021) and informing culturally tailored interventions for specific subgroups (Caughy et al., 2023; Chen et al., 2021).

Using person‐centered methodology, studies with Asian American adolescents have found that adolescents in profiles with high levels of cultural socialization (analogous to maintenance of heritage culture) messages consistently demonstrate better adolescent well‐being and ethnic identity development, particularly when negative ERS messages are low (Kiang et al., 2018; Xie et al., 2021). In particular, Kiang et al. (2018) found that profiles characterized by higher frequencies of preparation for bias, cultural socialization, and promotion of mistrust messages demonstrated more positive mental health outcomes compared to profiles with lower frequencies of the ERS messages (Kiang et al., 2018). However, promotion of mistrust messages appeared negative: profiles that combined high levels of preparation for bias and cultural socialization messages with low levels of promotion of mistrust messages demonstrated the most positive youth outcomes. Moreover, profiles with higher levels of promotion of mistrust messages were associated with diminished youth ethnic identity exploration. Promotion of mistrust messages heightens children's feelings of fear and distress, leading them to be cautious of individuals outside their ethnic–racial group (Kiang et al., 2018). Such emotions may conflict with identity exploration because the unfamiliar is perceived as threatening.

Similarly, Xie et al. (2021) found that adolescents in profiles with high levels of cultural socialization and low levels of preparation for bias messages demonstrated the most positive mental health and school engagement outcomes, while adolescents in the profile with low levels of cultural socialization and high levels of preparation for bias demonstrated the least positive mental health outcomes. This contrasts with Kiang et al. (2018), who found preparation for bias to be beneficial when not coupled with promotion of mistrust. As previous research has indicated mixed findings regarding the effect of preparation for bias messages on adolescent social–emotional well‐being and academic achievement, the positive or negative influence of preparation for bias may depend on the context (Wang, Henry, et al., 2020; Wang, Smith, et al., 2020).

Thus, parents' engagement in the cultural socialization of ERS messages plays a vital role in fostering resilience among Asian American youth when faced with racial discrimination and stereotyping. The benefits of positive ERS practices are maintained regardless of the frequency and severity of adolescents' discriminatory experiences (Kiang et al., 2018), highlighting the protective importance of positive ERS messages in supporting Asian American youth development. Notably, Kiang et al. (2018; three ERS subscales: cultural socialization, preparation for bias, promotion of mistrust) and Xie et al. (2021; two ERS subscales: cultural socialization and preparation for bias) did not assess Asian American ERS practices along all seven theoretically grounded ERS domains identified by Juang et al. (2016). Furthermore, these two studies used youth reports to measure parental ERS and youth outcomes, introducing mono‐method and shared‐method bias. Our study addresses these limitations by incorporating a more comprehensive measurement of ERS and by using both parent and adolescent ERS reports.

### Discrepancies in parent‐youth ERS reports

While few studies have examined discrepancies between parent and youth reports of ERS, existing research indicates a pattern of discordance between parent‐ and youth‐reported ERS that may be developmentally consequential (Caughy et al., 2023; Chen et al., 2021; Peck et al., 2014). This disconnect between parents' intended messages and youth's perception may stem from parents' unclear communication of messages, youths' inability to comprehend messages, and broader relational difficulties. Socialization mechanisms may also be implicit and nonverbal, leading to perceptual differences between intended and received parental ERS messages (Wang, Henry, et al., 2020). These differences are developmentally important as adolescents may miss out on positive ERS messages that could otherwise support their ability to cope with discrimination, socioemotional well‐being, or ethnic identity development.

Indeed, research has shown that discrepancies in ERS, especially when parents report higher levels of positive ERS than youth, are associated with poorer socioemotional, academic, and ethnic identity outcomes (Caughy et al., 2023; Chen et al., 2021). Conversely, in profiles where parent‐youth dyads report similarly high levels of ERS, youth demonstrate more positive mental health, academic, and ethnic identity outcomes (Caughy et al., 2023; Chen et al., 2021). When discrepancies exist, they are more pronounced for preparation for bias messages compared to cultural socialization messages (Caughy et al., 2023; Peck et al., 2014). These studies suggest that while parents believe they are engaging in preparation for bias, adolescents are often not perceiving or internalizing these messages. Discrepancies in preparation for bias also appear to be more consequential for youth development compared to cultural socialization. Using latent difference scores and structural equation modeling with Black and Latinx families, Caughy et al. (2023) found that parent‐youth discrepancies in cultural socialization reports were unrelated to adolescents' ethnic–racial identity development, while discrepancies in preparation for bias reports were negatively associated with all aspects of ethnic–racial identity.

In studies that have included both parent and child reports, the comparative significance of these two reports for adolescent development remains unclear. When Caughy et al. (2023) examined parent‐reported ERS practices alone, higher levels of cultural socialization and preparation for bias messages were positively associated with greater youth ethnic–racial identity development. However, profiles identified from Black American families using youth‐reported ERS were more directly linked to adolescent outcomes compared to those identified using parent‐reported ERS (Peck et al., 2014), suggesting that youth's perception and internalization of ERS messages is central to how ERS impacts their developmental outcomes. In the Peck et al. (2014) study, only adolescent‐reported cultural socialization ERS messages were directly related to adolescent racial identity development. Parent‐reported ERS was indirectly linked to adolescent racial identity, mediated by adolescent‐reported ERS. This illustrates the importance of parent–child concordance in the process of ERS transmission, as cultural socialization messages only produced benefits when both parent‐ and youth‐reported high levels of cultural socialization and not when parent‐reported high levels and youth‐reported low levels. This highlights youth's perception and internalization of messages as central to the effective transmission of ERS and indicates that high parent–child concordance may reflect high‐quality parent–child relationships characterized by effective communication, which can encourage youth to engage with potentially difficult conversations about culture and discrimination.

### The present study

While the benefits of ERS for Asian American adolescents have been increasingly recognized in developmental research, methodological and conceptual limitations persist in the literature. Prior studies have predominantly focused on a limited subset of ERS dimensions and relied on single‐informant designs, failing to capture meaningful information from both parents and youth (Caughy et al., 2023; Chen et al., 2021; Peck et al., 2014). Parent reports reflect intended ERS messages while adolescent reports capture perceived and/or internalized messages (Peck et al., 2014). Discrepancies between reporters provide information beyond either informant alone, capturing valuable context around interactions (De Los Reyes & Ohannessian, 2016). Using LPA enabled us to identify different patterns of parent‐adolescent dyadic reports of ERS and link these specific profiles to key developmental outcomes. The present investigation addressed these limitations by examining dyadic parent‐adolescent perspectives on all seven dimensions of ERS practices in Asian American families defined by Juang et al. (2016). We employed LPA to identify patterns of parent and adolescent ERS reports based on both content (absolute levels of seven positive and negative ERS domains) and process (degree of parent‐adolescent agreement). The study had two primary aims: (1) to identify distinct profiles of parent‐adolescent ERS patterns using LPA and (2) to examine associations between identified ERS profiles and adolescent adjustment outcomes, including mental health, academic performance, social competence, and ethnic identity.

Based on prior work, we hypothesized the emergence of several distinct profiles characterized by varying levels of positive and negative ERS messages and parent‐adolescent agreement. It was also hypothesized that profiles characterized by higher levels of positive ERS and lower levels of negative ERS, and higher levels of parent‐adolescent agreement would be associated with the most favorable youth adjustment outcomes across mental health, academic, social, and ethnic identity. Conversely, it was expected that profiles displaying lower levels of positive ERS, higher levels of negative ERS, and lower levels of agreement would be associated with poorer adjustment outcomes. Both the integrative model (García Coll et al., 1996) and Mistry and Kiyama's (2021) framework position family communication and shared understanding around ERS as mechanisms through which cultural messages can impact youth development (García Coll et al., 1996; Mistry & Kiyama, 2021). Parent‐adolescent agreement may be an indicator of effective communication and understanding, reflecting the quality of these meaning‐making processes. Thus, distinct patterns of ERS should meaningfully differentiate families, and both what is communicated (positive versus negative messages) and how effectively it is transmitted (parent‐adolescent agreement) may shape developmental competencies in Asian American youth.

## METHODS

### Participants

The analytic sample comprised 466 Asian American parent‐adolescent dyads, including 317 Chinese American, 118 Korean American, and 31 Filipino American dyads. Parents were ages 30–65 (*M*_age_ = 46.52 years, *SD* = 5.21), and their adolescents were ages 9–19 (*M*_age_ = 14.72 years, *SD* = 1.91). The majority of parents (87%) reported having a college degree or higher. The median household income was in the $100–149,999 range. Most parents (94%) were born outside of the United States, and most adolescents (80%) were born in the United States (2nd generation). Most parents (81%) were mothers, and the majority of parents (89%) were married. At the time of this study, participants resided across 30 U.S. states, with the largest concentrations in Maryland (35%), California (14%), New York (12%), and Virginia (11%). Adolescents were 232 boys and 232 girls, with 2 undisclosed gender. No significant differences in demographic data were found across the three ethnic subgroups. Data from the present study were obtained from the first wave of a longitudinal study examining Asian American immigrant families' adjustment during the COVID‐19 pandemic.

### Procedure

Participants were recruited from Asian American community organizations, language and cultural schools, religious centers, and social media groups through the distribution of e‐flyers via emails, texts, or phone calls. Interested families initially received links to the online screening hosted by the Qualtrics platform to determine their eligibility (e.g., race–ethnicity, age of children). Participants had to self‐ identify as Chinese/Chinese American, Filipino/Filipino American, or Korean/Korean American. Parents first provided online consent for themselves and their adolescent's participation, followed by adolescents' online assent via a separate link. Eligible parents and their adolescents received separate survey links. Survey data collection occurred between 2022 and 2023. To ensure the validity of the data, we reviewed each parent–child dyad's survey upon completion. When a substantial amount of data was missing, we contacted the dyad to verify their responses and invited them to complete any missing items. Participants exhibiting repetitive response patterns were contacted by phone or email to confirm their entries, and those successfully reached were responsive. Surveys completed in an unusually short time, based on pilot data and sample‐wide completion norms, or containing self‐contradictory information were excluded from the final dataset. Measures were available in English, simplified Chinese, traditional Chinese, Tagalog, and Korean, using the back‐translation method. Families received $35 e‐gift cards as compensation for their time. Ethical approval for this study was obtained from the University of Maryland Institutional Review Board (Approval Number: Y16CC20229).

### Measures

#### Demographics

Parents provided demographic information, including each parent's and their adolescents' age, gender, place of birth, years in the United States, marital status, education level, and household income in 2020. Race and ethnicity were assessed with a single‐selection item asking participants to indicate the category that best described them (Chinese/Korean/Filipino, Asian, Chinese/Korean/Filipino American, Asian American, American, or Other with a write‐in option). This item was customized based on the survey version that families received at recruitment. For example, participants completing the Chinese version of the survey selected from the following options: Chinese, Chinese American, Asian American, American, or Other (write‐in). This item was used to capture participants' preferred self‐description but was not used to determine study eligibility.

#### Asian American ethnic–racial socialization

Adolescents and their parents separately completed the *Asian American Parental Racial*–*Ethnic Socialization Scale* (Juang et al., 2016), measuring parents' frequency of using seven ERS practices: maintenance of heritage culture, becoming American, awareness of discrimination, promotion of equality, cultural pluralism, avoidance of outgroups, and minimization of race. Each ERS subscale was measured on a 1–5 Likert scale (1 = *never* to 5 = *very often*). The mean score was calculated for each subscale, with higher scores indicating parents' more frequent communication of the ERS practice.

The maintenance of heritage culture subscale (8 items; e.g., “How often did you routinely cook Asian food for your children?”) assessed parents' efforts to teach children about their cultural heritage, traditions, and language. The Cronbach's α was .76 for both parents and adolescents in our sample. Cultural pluralism (4 items; e.g., “How often did you encourage your children to have friends from other racial/ethnic backgrounds?”) evaluated parents' encouragement of diversity and cross‐cultural relationships. The Cronbach's α was .85 for both parents and adolescents in our sample. Promotion of equality (3 items; e.g., “How often did you show your children that all people are equal regardless of race or ethnicity?”) measured parents' emphasis on treating all people equally regardless of racial/ethnic background. The Cronbach's α was .81 for parents and .83 for adolescents in our sample. Becoming American (4 items; e.g., “How often did you tell your children to have close friends who were (non‐Asian) Americans?”) measured parents' encouragement of children to integrate into mainstream American society. The Cronbach's α was .73 for parents and .64 for adolescents in our sample.

Awareness of discrimination (3 items; e.g., “How often did you talk to your children about why some people will treat them unfairly because of their Asian background?”) evaluated parents' discussions with children about discrimination they might face due to their ethnicity. The Cronbach's α was .78 for parents and .80 for adolescents in our sample. Avoidance of outgroups (4 items; e.g., “How often did you tell your children to avoid another racial or ethnic group?”) measured parents' messaging about avoiding contact with people from other racial/ethnic groups. The Cronbach's α was .91 for parents and .90 for adolescents in our sample. Minimization of race (3 items; e.g., “How often did you give your children the impression that issues of race and racism were not important?”) assessed parents' tendency to downplay the significance of race or racism. The Cronbach's α was .79 for both parents and adolescents in our sample.

#### Mental health

Adolescents completed the *Generalized Anxiety Disorder 7‐Item Scale* (GAD‐7; Spitzer et al., 2006), which measured anxiety symptoms. Adolescents rated how frequently they experienced symptoms over the past 2 weeks on sample items such as “feeling nervous, anxious, or on edge” and “feeling afraid as if something awful might happen.” The items were measured on a 4‐point scale (1 = *not at all sure* to 4 = *nearly every day*). The mean composite score represents anxiety symptoms, with a higher score indicating higher levels of anxiety. The Cronbach's α for adolescents was .97 for this sample.

Adolescents also completed the *Patient Health Questionnaire‐9* (PHQ‐9; Kroenke et al., 2001), which measured depressive symptoms. Adolescents reported how frequently they experienced various symptoms (e.g., “feeling down, depressed, or hopeless,” “little interest or pleasure in doing things”) over the past 2 weeks using a 4‐point scale (0 = *not at all sure* to 3 = *nearly every day*). The mean composite score represents depressive symptoms, with higher scores indicating higher levels of depression. The Cronbach's α was .95 for this sample.

#### Academic performance

Adolescents reported their performance in academic subjects, including English or language arts, arithmetic or math, history or social studies, and science. Their academic performance for each subject was measured on a 4‐point scale (1 = *failing* to 4 = *above average*). The mean composite score was calculated to represent their overall academic performance, with higher scores indicating better academic performance.

#### Social competence

Adolescents' social competence was assessed using six items from the *Profiles of Student Life‐Attitudes and Behaviors* scale (Scales et al., 2000). Adolescents rated their agreement with statements about their social abilities (e.g., “I get along well with my friends,” “I resolve conflicts peacefully”) on a 5‐point scale (1 = *strongly disagree* to 5 = *strongly agree*). The mean composite score represents social competence, with higher scores indicating greater social competence. The Cronbach's α was .73 in this sample.

#### Ethnic identity and American identity

Adolescents completed adapted subscales from the *Multidimensional Inventory of Black Identity* (MIBI; Sellers et al., 1997) to assess four different identities: ethnic identity (Chinese/Korean/Filipino), ethnic American identity (Chinese/Korean/Filipino American), Asian American identity, and American identity. Adolescents received a Chinese, Korean, or Filipino version of the ethnic identity and ethnic American identity measure depending on how they self‐identified. Two subscales were used: centrality (5 items; e.g., “In general, being ____ is an important part of my self‐image”) and private regard (6 items; e.g., “I feel good about ____ people”). Items were rated on a 5‐point scale (1 = *strongly disagree* to 5 = *strongly agree*). Mean composite scores were calculated separately for centrality and private regard for each identification group, with higher scores indicating stronger identification and more positive feelings toward the group, respectively. Cronbach's α values ranged from .80 to .85 for centrality and .71 to .89 for private regard.

### Analysis Plan

Latent profile analyses were conducted using Mplus 7.4 (Muthén & Muthén, 1998) for parent‐adolescent dyads to find patterns of parental ERS based on parent and youth reports. All seven ERS indicators were included: maintenance of heritage culture, cultural pluralism, promotion of equality, becoming American, awareness of discrimination, avoidance of outgroups, and minimization of race. ERS indicators were standardized as z‐scores prior to running the LPA. Missing data ranged from 0.43% to 2.36% across LPA indicators and were handled using Full Information Maximum Likelihood (FIML) estimation. We examined solutions ranging from one to six profiles. Model fit indices were used to determine the optimal number of profiles, including the Akaike Information Criterion (AIC), the Bayesian Information Criterion (BIC), sample size–adjusted BIC (SABIC), and the bootstrapped likelihood ratio test (BLRT). Decision criteria included smaller AIC, BIC, and SABIC values, a statistically significant BLRT, and improved interpretability of profiles. An optimal solution was determined at the point where decreases in these values began to level off with the addition of more profiles. Model entropy was also examined, with values above .80 considered indicative of good classification (Muthén, 2007). After identifying the optimal profile solution, we used the BCH command (Nylund‐Gibson et al., 2019) to examine mean‐level differences between profiles on Asian American youth adjustment (social competence, perceived character, academic grades, and ethnic identity).

## RESULTS

### Descriptive statistics

Across all parent‐adolescent data, mean levels of ERS ranged from 1.50 to 3.83 on a 1 to 5 scale. Correlations between parent and adolescent reports of each ERS subscale ranged from *r* = .18 to *r* = .45, with promotion of equality demonstrating the lowest parent‐adolescent correspondence and maintenance of heritage culture demonstrating the highest. Maintenance of heritage culture was the most common form of socialization reported by both parent and adolescent (*M*_parent_ = 3.76, *M*_adolescent_ = 3.83), followed by promotion of equality (*M*_parent_ = 3.58, *M*_adolescent_ = 3.27), cultural pluralism (*M*_parent_ = 3.27, *M*_adolescent_ = 2.95), becoming American (*M*_parent_ = 2.89, *M*_adolescent_ = 2.65), awareness of discrimination (*M*_parent_ = 2.61, *M*_adolescent_ = 2.59), minimization of race (*M*_parent_ = 1.57, *M*_adolescent_ = 1.69), and avoidance of outgroups (*M*_parent_ = 1.50, *M*_adolescent_ = 1.72). Bivariate correlations among study variables are presented in Tables 1 and 2.

**TABLE 1:** Correlations among ERS variables.

Variable	1	2	3	4	5	6	7	8	9	10	11	12	13	14
1. Parent: Heritage Culture Maintenance		.45	.22	.04	.24	.14	.25	.07	.01	−.02	−.06	−.06	.19	.04
2. Youth: Heritage Culture Maintenance			−.04	.11	.03	.16	.10	.16	−.04	−.05	−.08	−.14	.08	.20
3. Parent: Becoming American				.31	.39	.13	.48	.22	.31	.13	.27	.15	.25	.08
4. Youth: Becoming American					.23	.35	.19	.49	.16	.20	.06	.25	.07	
5. Parent: Awareness of Discrimination						.35	.35	.17	.41	.18	.24	.14	.18	.04
6. Youth: Awareness of Discrimination							.14	.30	.20	.43	.08	.21	.01	.10
7. Parent: Cultural Pluralism								.26	.06	−.04	.05	−.02	.66	.17
8. Youth: Cultural Pluralism									−.02	−.07	−.01	.04	.17	.67
9. Parent: Avoidance of Outgroups										.35	.70	.29	−.10	−.15
10. Youth: Avoidance of Outgroups											.23	.62	−.14	−.20
11. Parent: Minimization of Race												.26	−.00	−.05
12. Youth: Minimization of Race													−.10	−.04
13. Parent: Promotion of Equality														.18
14. Youth: Promotion of Equality														

*Note*: Values represent Pearson zero‐order correlations among parent‐ and youth‐reported ethnic–racial socialization (ERS) indicators. Higher scores indicate greater endorsement of each practice.

**TABLE 2:** Correlations among ERS variables and youth outcome variables.

Variable	15	16	17	18	19	20
1. Parent: Heritage Culture Maintenance	−.04	−.05	.01	.05	−.03	.06
2. Youth: Heritage Culture Maintenance	−.02	−.05	.11	.24	.02	.17
3. Parent: Becoming American	−.04	−.06	−.03	.07	−.04	−.08
4. Youth: Becoming American	.00	.00	.00	.09	.12	−.03
5. Parent: Awareness of Discrimination	.07	.09	−.08	−.05	−.05	−.07
6. Youth: Awareness of Discrimination	.24	.24	−.04	.08	−.06	−.06
7. Parent: Cultural Pluralism	−.02	−.01	−.02	.07	.00	−.02
8. Youth: Cultural Pluralism	−.06	−.07	.05	.15	.18	.12
9. Parent: Avoidance of Outgroups	.04	.11	−.18	−.13	−.07	−.11
10. Youth: Avoidance of Outgroups	.36	.38	−.16	−.07	−.21	−.17
11. Parent: Minimization of Race	.05	.09	−.15	−.06	−.03	−.12
12. Youth: Minimization of Race	.17	.23	−.11	−.05	−.14	−.18
13. Parent: Promotion of Equality	−.00	.00	−.00	.09	.05	.02
14. Youth: Promotion of Equality	−.08	−.10	.05	.20	.25	.17
15. Anxiety Symptoms		.83	−.17	−.13	−.17	−.07
16. Depressive Symptoms			−.26	−.18	−.25	−.09
17. Academic Performance				.27	.05	.10
18. Social Competence					.18	.19
19. American Identity Private Regard						.27
20. Ethnic Identity Private Regard						

*Note*: Values represent Pearson zero‐order correlations between ERS indicators and youth adjustment outcomes. Higher scores reflect greater endorsement of each construct. Correlations are based on pairwise complete observations.

### LPA model fit

Analysis of ERS profiles yielded an optimal four‐profile solution (Table 3). The BIC (16032.07) and SABIC (15816.25) showed diminishing improvements beyond four profiles. For instance, the BIC decreased modestly from the four‐ to five‐profile model (16032.07–15978.25) and again from the five‐ to six‐profile model (15978.25–15882.47). The BLRT was statistically significant (*p* < .001), further supporting this solution. The model entropy value peaked at four profiles, and a value of .86 indicated good classification accuracy. Regarding profile sizes, the smallest profile contained 24 cases (5% of the sample), suggesting adequate profile sizes for interpretation. As the number of profiles increased, the smallest class size continued to decrease, leading to inadequate sample sizes for the models with a higher number of profiles. Therefore, the four‐profile solution was selected.

**TABLE 3:** Latent profile model fit indices.

Number of profiles	AIC	BIC	Sample size–adjusted BIC	Entropy	Bootstrapped LRT	Bootstrapped LRT *p*	Class sizes
1	17034.18	17156.92	17064.06	1.00	N/A	N/A	466
2	16521.28	16758.14	16593.80	.74	484.62	<.001	245, 221
3	16055.73	16313.59	16118.47	.83	491.97	<.001	195, 173, 98
4	15750.26	16032.07	15816.25	.87	335.32	<.001	194, 159, 89, 24
5	15604.87	15978.25	15677.69	.84	120.36	<.001	145, 132, 86, 80, 23
6	15475.16	15882.47	15553.81	.86	146.58	<.001	146, 126, 76, 63, 41, 14

*Note*: Entropy values closer to 1 indicate better classification quality. Bootstrapped LRT = likelihood ratio test comparing k versus k–1 class model.

Abbreviations: AIC, Akaike information criterion; BIC, Bayesian information criterion.

### Profile characteristics

**FIGURE 1:**
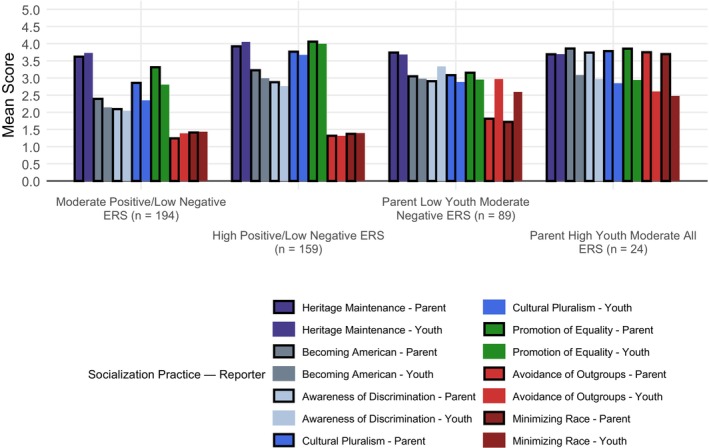
Standardized parent‐ and adolescent‐reported ers practices by profile. Bars are colored by ERS practice. Parent‐reported ERS practices have black outlines; youth‐reported ERS practices have no outline.

The profiles captured distinct patterns across the seven ERS dimensions, as shown in Figure 1 and Figure S1. Profiles were named based on the relative frequency of positive and negative ERS messages and the degree of parent‐adolescent agreement, compared across profiles. Frequencies of positive or negative ERS practices were designated to be low (mean score < 2), moderate (2.5 < mean score < 3.5), or high (mean score > 3.5). Profiles were labeled (a) Moderate Positive/Low Negative ERS (40%, *n* = 182); (b) High Positive/Low Negative ERS (36%, *n* = 163); (c) Parent Low Youth Moderate Negative ERS (19%, *n* = 85); and (d) Parent High Youth Moderate All ERS (5%, *n* = 23). The first and largest profile, Moderate Positive/Low Negative ERS, had overall moderate reports of positive ERS and low negative ERS from parents and youth with moderate‐high agreement. Parents and youth in this profile reported lower scores compared to other profiles on becoming American, awareness of discrimination, and cultural pluralism. While agreement was high overall, parents reported somewhat higher levels of cultural pluralism and promotion of equality messages compared to youth.

The second profile, High Positive/Low Negative ERS, displayed high parent and youth reports of positive ERS and low negative ERS. Adolescent reports of cultural pluralism and promotion of equality messages were particularly high compared to other profiles. This profile also demonstrated the strongest parent‐adolescent concordance across all ERS dimensions. The third profile, Parent Low Youth Moderate Negative ERS, was characterized by youth reporting higher levels of negative ERS compared to parents, with notable discrepancies in parent‐adolescent reports of avoidance of outgroups and minimization of race. While parents and youth both reported overall moderate levels of positive ERS, adolescents reported higher levels of awareness of discrimination and lower levels of cultural pluralism and promotion of equality compared to parents. The final and smallest profile, Parent High Youth Moderate All ERS, was distinguished by parents reporting higher levels of both positive and negative ERS dimensions, with substantial parent‐adolescent discrepancies for all ERS dimensions except maintenance of heritage culture. This profile displayed the highest parent‐reported becoming American, awareness of discrimination, avoidance of outgroups, and minimization of race.

### Profile differences in youth outcomes

Results revealed several significant differences in youth outcomes across the four ethnic–racial socialization profiles (Table 4). Regarding mental health outcomes, youth in the two profiles with low negative ERS and high agreement (High Positive/Low Negative ERS and Moderate Positive/Low Negative ERS) displayed significantly lower levels of anxiety and depression symptoms compared to youth in the Parent Low Youth Moderate Negative ERS profile. Specifically, youth in the Parent Low Youth Moderate Negative ERS profile reported significantly higher GAD‐7 symptoms (*M* = 1.77) than youth in the Moderate Positive/Low Negative ERS profile (*M* = 1.28) and the High Positive/Low Negative ERS profile (*M* = 1.26). The 0.49–0.51 mean‐score differences between these groups correspond to a difference of approximately 3.4–3.6 points on the GAD‐7 total score, which can represent clinically significant differences (e.g. between minimal and mild anxiety). For depression symptoms, youth in the Parent Low Youth Moderate Negative ERS profile reported higher PHQ‐9 scores (*M* = 1.57) than those in the Moderate Positive/Low Negative ERS (*M* = 1.13) and High Positive/Low Negative ERS (*M* = 1.08) profiles. These contrasts reflect approximately 3.9–4.4 point differences on the PHQ‐9 total score, differences that approach clinically meaningful change thresholds.

**TABLE 4:** Mean‐level profile differences in youth outcomes.

Outcome	Moderate positive/low negative ERS (42%)		High positive/low negative ERS (34%)		Parent low youth moderate negative ERS (19%)		Parent high youth moderate all ERS (5%)		Test statistics	
	M	SD	M	SD	M	SD	M	SD	χ^2^	*p*
GAD	1.28^a^	0.06	1.26^a^	0.07	1.77^b^	0.11	1.54^ab^	0.18	18.82	<.001
PHQ	1.13^a^	0.05	1.08^a^	0.05	1.57^b^	0.09	1.37^ab^	0.17	24.96	<.001
Academic performance	3.64^ab^	0.03	3.69^a^	0.04	3.53^bc^	0.06	3.30^c^	0.15	10.82	0.013
Social competence	3.86^a^	0.04	4.08^b^	0.04	3.87^a^	0.06	3.69^a^	0.14	17.79	<.001
American Identity Private Regard	3.63^a^	0.07	3.85^b^	0.07	3.35^a^	0.15	3.28^a^	0.20	14.80	0.002
Asian American Identity private regard	4.00^ab^	0.05	4.15^a^	0.06	3.86^b^	0.10	3.80^ab^	0.20	8.29	0.040
Chinese/Korean/Filipino American Identity Private Regard	4.10^b^	0.05	4.28^a^	0.06	3.90^b^	0.11	3.99^ab^	0.16	11.86	0.008
Chinese/Korean/Filipino Identity Private Regard	4.10^a^	0.06	4.28^a^	0.10	3.80^b^	0.11	3.40^b^	0.25	18.49	<.001

*Note*: Means that share a superscript letter do not differ significantly based on BCH post‐hoc tests (*p* > .05). Some groups share multiple superscripts due to overlapping non‐significant relationships.

Academic performance also varied meaningfully across profiles, with youth in the High Positive/Low Negative ERS profile demonstrating significantly better academic outcomes compared to youth in the two profiles with higher negative ERS and low agreement (Parent High Youth Moderate All ERS and Parent Low Youth Moderate Negative ERS). Youth in the Moderate Positive/Low Negative ERS profile also had better academic outcomes compared to youth in the Parent High Youth Moderate All ERS profile. Furthermore, adolescents' social competence varied across profiles. Youth in the High Positive/Low Negative ERS profile scored significantly higher on social competence compared to youth in all other profiles.

Ethnic identity private regard also revealed differences across different profiles. Youth in the High Positive/Low Negative ERS profile reported significantly higher levels of American identity private regard compared to youth in all other profiles. For Asian American private regard, youth in the High Positive/Low Negative ERS profile reported significantly higher levels of private regard compared to those in the Parent Low Youth Moderate Negative ERS profile. Regarding their private regard of their ethnic American identity (e.g., Chinese American, Korean American, or Filipino American identity), youth in the High Positive/Low Negative ERS profile reported significantly higher levels compared to the Moderate Positive/Low Negative ERS and Parent Low Youth Moderate Negative ERS profiles. Regarding heritage ethnic identity private regard (e.g., Chinese, Korean, or Filipino identity), youth in the two profiles with low negative ERS and high agreement (High Positive/Low Negative ERS and Moderate Positive/Low Negative ERS) showed significantly higher levels than youth in the two profiles with higher negative ERS and low agreement (Parent Low Youth Moderate Negative ERS and the Parent High Youth Moderate All ERS).

## DISCUSSION

Parental ERS in Asian American families plays an essential role in shaping adolescents' cultural identity, psychological adjustment, and resilience in navigating multicultural contexts (Cheah et al., 2021; Park et al., 2021). Within this complex process, adolescents may internalize, misinterpret, or ignore various parental ERS messages. However, no study has explored discrepancies between parent and adolescent perceptions of parents' ERS practices. The present study extended prior research by investigating patterns of parents' and adolescents' perceived frequencies of parents' ERS practices, and parent‐adolescent agreement on these practices, examining how these socialization patterns were associated with youth adjustment.

### Patterns of parent‐adolescent ERS reports

Our findings revealed significant variability in how Asian American parents and adolescents perceive parent ERS practices within their families. Parent and adolescent reports vary in how they converge or diverge; 76% of the dyads in our sample displayed converging patterns of ERS reports with high agreement. The largest profile (42% of our sample) was characterized by moderate levels of positive ERS and low levels of negative ERS practices, while the second‐largest profile (34%) featured high levels of positive ERS and low levels of negative ERS practices, with both profiles displaying high parent‐adolescent agreement. These two profiles suggest that most parents and adolescents had similar perceptions of the nature and frequency of parental ERS practices. This convergence in parent‐adolescent perspectives can serve as a proxy for family functioning, reflecting shared understanding and effective communication within families (De Los Reyes & Ohannessian, 2016). It should be noted that due to our predominantly high‐SES and college educated sample, the high proportion of concordant dyads may reflect contexts and greater access to resources that support more positive parent–child communication.

Conversely, 24% of dyads displayed diverging patterns of ERS practices reports with low agreement. The third profile (19%) was distinguished by adolescents reporting that their parents engaged in higher levels of negative ERS practices than their parents reported, particularly regarding avoidance of outgroups and minimization of race messages. These discrepancies may be reflective of communication gaps that result from parents not effectively communicating, adolescents interpreting parents' ERS through the lens of their unique experiences, or adolescents misinterpreting parents' ERS messages. Parents may also be unintentionally communicating attitudes that encourage separation from other racial/ethnic groups or that downplay the importance of race through comments about other ethnic groups, reactions to racial issues, or implicit behaviors that demonstrate discomfort with other racial/ethnic groups. Adolescents may also have different interpretations of ERS messages from their parents due to adolescents' exposure to multicultural peer environments and education on racial dynamics (Patel et al., 2023), allowing adolescents to more easily recognize negative ERS messages.

Finally, the smallest profile (5%) featured parents reporting higher levels of both positive and negative ERS practices than did their adolescents, with particularly large discrepancies on messages about becoming American, awareness of discrimination, avoidance of outgroups, and minimization of race. This pattern indicates another potential communication breakdown, where parents believe they are actively transmitting ERS messages to their adolescents, but these messages are not being registered by their adolescents at the same level. This finding aligns with previous research with Mexican‐origin (Chen et al., 2021) and Black American families (Peck et al., 2014), indicating that parents often report delivering more frequent ERS messages than their children perceive receiving. Parents may be communicating ERS messages in ways that do not resonate with adolescents' experiences, or parents may be reporting intended levels of ERS that differ from reality.

Consistent with previous findings highlighting the importance of cultural socialization in Asian American families (Atkin & Yoo, 2021; Juang et al., 2016; Ren et al., 2022), maintenance of heritage culture emerged as the most frequently reported form of ERS across all profiles. Asian American youth report receiving these messages more frequently than other ERS messages (Atkin & Yoo, 2021; Ren et al., 2022), potentially attributable to parents' consistent delivery of these messages through daily cultural practices and cultural events (Atkin & Yoo, 2021; Juang et al., 2016). Parent‐adolescent agreement was also highest for maintenance of heritage culture, similar to Caughy et al.'s (2023) finding that cultural socialization was a more agreed upon ERS dimension compared to preparation for bias among Black and Latinx samples. In contrast, promotion of equality yielded the lowest parent‐adolescent concordance, potentially reflecting more subtle communication about equality that adolescents may not recognize as intentional ERS. Parents may need to communicate these values more explicitly by directly discussing them, providing and modeling concrete examples of egalitarian behaviors, or actively engaging their youth in activities that emphasize equality.

The emergence of these four different profiles further extends both García Coll et al.'s (1996) and Mistry and Kiyama's (2021) theoretical frameworks, demonstrating that Asian American families have diverse configurations of ERS practices that reflect unique social contexts, resources, and adaptive strategies. Differences in profile outcomes may be interpreted through Mistry and Kiyama's (2021) framework: profiles characterized by higher positive ERS and stronger parent‐adolescent agreement may represent families engaging in strong, collaborative meaning‐making that enables youth to construct alternative narratives and counter pervasive negative stereotypes through positive cultural affirmation. The development of these unified narratives can also promote youth's understanding of their ethnic–racial identity, preparing them to navigate marginalization. On the other hand, profiles with lower agreement and higher negative ERS may reflect families struggling to establish shared narratives, leaving youth more vulnerable to marginalization.

### ERS profiles and adolescent adjustment

Different parent‐adolescent profiles were meaningfully associated with adolescents' mental health, academic performance, social competence, and ethnic identity, providing insights into how both perceived frequencies of positive and negative ERS and parent‐adolescent agreement in these perceptions shape youth development. These associations support the integrative model's (García Coll et al., 1996) proposition that family processes function as adaptive mechanisms for cultivating developmental competencies in minoritized youth. They also extend Mistry and Kiyama's (2021) framing of collaborative parent–child meaning‐making and youth ethnic–racial identity development as key to youth's successful navigation of marginalization. Our findings suggest that both the content of ERS messages and the convergence of parent‐youth perceptions of ERS are critical for shaping youth development.

First, adolescents in profiles with higher levels of positive ERS and lower levels of negative ERS demonstrated better adjustment outcomes. Adolescents in the profile marked by parent and adolescent reports of frequent positive ERS and infrequent negative ERS (High Positive/Low Negative ERS) demonstrated the most positive outcomes across mental health, academic achievement, social competence, and ethnic identity private regard. Consistent differences emerged between these adolescents and those who reported that their parent used more negative ERS messages (Parent Low Youth Moderate Negative ERS and Parent High Youth Moderate All ERS). Furthermore, adolescents reporting high positive ERS and low negative ERS (High Positive/Low Negative ERS) from their parents were not only more socially competent compared to adolescents who reported more frequent negative ERS, but also compared to adolescents who reported infrequent negative ERS but only moderate levels of positive ERS (Moderate Positive/Low Negative ERS).

In alignment with the integrative model's (García Coll et al., 1996) emphasis on how family processes (e.g., ERS) can cultivate developmental competencies, the nature and frequency of ERS messages can promote or undermine youth adjustment. Our findings are consistent with prior research showing that parents' engagement in more positive ERS practices may increase youth ethnic pride, cultural knowledge, and bicultural competence, promoting their mental health (Atkin et al., 2019; Park et al., 2021), academic performance (Wang, Smith, et al., 2020), and social competence (Tran & Lee, 2010; Wang, Henry, et al., 2020). In contrast, more frequent negative ERS practices may lead to outgroup avoidance or identity confusion that could negatively impact youth adjustment (Atkin et al., 2019; Park et al., 2021).

Second, profiles combining higher parent‐adolescent agreement with high positive and low negative ERS displayed better youth adjustment. Adolescents in the profile with the highest parent‐adolescent agreement (High Positive/Low Negative ERS) displayed more adaptive functioning across mental health, academic performance, social competence, and ethnic identity development compared to profiles with lower parent‐adolescent agreement (Parent Low Youth Moderate Negative ERS, Parent High Youth Moderate All ERS). High levels of parent‐adolescent concordance may reflect a shared understanding and more effective or clear communication, contributing to an environment that supports positive adjustment. Repeated research has linked effective parent–child communication and supportive parent‐adolescent relationships with adolescent emotional and behavioral adjustment (Ratliff et al., 2023). Positive parent‐adolescent communication protects children's well‐being and reduces behavioral problems (Guilamo‐Ramos et al., 2006). Close parent‐adolescent relationships have also been associated with favorable long‐term outcomes (e.g., lower depressive symptoms, stress, less substance abuse, greater optimism, and better romantic relationships) extending from adolescence to young adulthood (Ford et al., 2023). Drawing on these findings, we conceptualized parent‐adolescent agreement as an indicator of the effectiveness with which ERS messages were communicated, interpreted, and integrated in the family system.

In contrast, adolescents who reported higher levels of negative ERS than their parents (Parent Low Youth Moderate Negative ERS) demonstrated significantly poorer mental health, academic, social competence, and ethnic identity outcomes compared to adolescents in profiles with low negative ERS and higher parent‐adolescent agreement. This suggests that families with lower parent‐adolescent agreement and higher youth‐perceived negative ERS have youth who experience greater psychological distress, have more academic difficulties, and may be less likely to access support from their co‐ethnic community due to weaker ethnic affiliations. Consistent with prior findings, higher levels of youth‐reported negative ERS messages are associated with increased youth mental health distress (Atkin et al., 2019; Park et al., 2021). Low agreement between youth and parents may signal misunderstanding at home or inconsistent socialization, leading to negative psychosocial adjustment outcomes (De Los Reyes & Ohannessian, 2016).

Even when profiles exhibited higher levels of parent‐ and youth‐reported positive ERS, when combined with lower levels of parent‐youth agreement, this overall pattern was associated with poorer outcomes. Adolescents in the profile with lower adolescent‐reported ERS compared to parent‐report ERS across all dimensions (Parent High Youth Moderate All ERS) exhibited poorer academic and ethnic identity outcomes compared to adolescents in profiles with higher parent‐adolescent agreement. Greater parent‐adolescent agreement may indicate more effective transmission and understanding of ERS messages, while discrepancies may signal misalignment in understanding or a weaker parent–child relationship. Consistent with Caughy et al.'s (2023) finding that Black and Latinx adolescents who reported lower levels of preparation for bias than their parents had poorer adolescent academic and identity outcomes, youth who reported lower levels of ERS compared to their parents exhibited poorer adjustment. When parents think that they are engaging in more ERS messages than adolescents perceive, these messages may not be effectively transmitted or processed to the extent that they are able to positively impact youth development (De Los Reyes & Ohannessian, 2016). The lack of alignment between parent and youth perceptions may underscore poor communication in the family, which has been linked to worse youth psychosocial adjustment (Xiao et al., 2011). However, it is worth noting that this profile was the smallest (5% of the sample), suggesting that very few Asian American parent‐youth dyads used high levels of both negative and positive ERS practices. Most Asian American parent‐adolescent dyads demonstrated adaptive ERS patterns for youth development.

Thus, differences across profiles suggest that the joint configuration of ERS content (positive and negative messages) and parent‐adolescent convergence may influence youth adjustment. The profile that exhibited the most positive outcomes combined higher levels of positive ERS, lower levels of negative ERS, and high levels of parent‐adolescent agreement, consistent with prior research with Mexican‐origin adolescents (Chen et al., 2021). Adolescents in profiles combining higher positive ERS, lower negative ERS, and higher parent‐adolescent agreement may be better equipped to make meaning of their ethnic–racial identity, construct empowering narratives, and cope with marginalization (Mistry & Kiyama, 2021). The findings also suggest that beyond ERS frequency, the degree of agreement between parents and youth may be a robust indicator of (a) adolescents' different perception/interpretation of the ERS practice at home and (b) strong parent‐child relationships and family functioning, beyond the accuracy of either party's perception. Prior research suggests that youth perception of parents' ERS practices is more strongly linked to youth adjustment than parent perception (Caughy et al., 2023). High agreement may also indicate a shared understanding of family values that promotes youth well‐being. Thus, our findings using a person‐centered approach imply that a more comprehensive understanding of socialization necessitates the simultaneous examination of the frequency of specific socialization messages and the level of agreement between both parties.

Our findings also help clarify ambiguity around the role of preparation for bias or awareness of discrimination messages on adolescent development. Consistent with Kiang et al. (2018), our results indicated that awareness of discrimination was associated with positive youth adjustment when communicated alongside higher levels of positive ERS and lower levels of negative ERS. However, in profiles that combined high levels of awareness of discrimination with higher levels of negative ERS (Parent High Youth Moderate All ERS), youth experienced poorer adjustment, including worse academic and ethnic identity outcomes. The context in which awareness of discrimination messages are delivered may influence whether those messages are protective, such as when communicated alongside affirming cultural messages, or harmful, such as when communicated in a fearful or isolating manner. This builds on findings that preparation for bias messages can be beneficial for adolescents when coupled with cultural socialization messages (Kiang et al., 2018) as youth can perceive these combined ERS practices as empowering for their identity development (Atkin et al., 2019). On the other hand, when preparation for bias is delivered in the absence of cultural socialization and/or coupled with negative ERS practices, this may place emphasis on threats without providing cultural resources that facilitate adaptive coping and identity development, leaving adolescents vulnerable to discrimination‐related stress (Xie et al., 2021). While our theoretical frameworks suggest that awareness of discrimination messages are important to allow youth to recognize potential barriers and marginalization, the context‐dependent effects of these messages add the nuance that ERS practices cannot be evaluated in isolation and must be understood in tandem.

### Limitations

Several limitations of the current study warrant acknowledgement. First, Asian Americans represent a highly heterogeneous population, encompassing numerous ethnic groups with distinct immigration histories, pre‐ and post‐migration experiences, acculturation trajectories, and cultural values (Mistry et al., 2016). These differences influence individuals' experiences of marginalization and their ERS practices in the United States (Mistry & Kiyama, 2021). Although often grouped under the pan‐ethnic label of “Asian American,” Chinese American, Korean American, and Filipino American families differ in their ethnic–racial socialization practices due to distinct immigration histories and racial positioning, particularly among first‐generation parents. For Chinese and Korean Americans, recent immigrants often include skilled professionals and students. Chinese and Korean language schools and cultural programs play an important role in the community to preserve linguistic and cultural heritage. Chinese American parents may emphasize heritage maintenance, achievement, and behavioral strategies to manage discrimination, with race‐related discussions often remaining indirect. Korean American parents' socialization is frequently shaped by strong ethnic institutions, leading to greater emphasis on preparation for bias, ethnic solidarity, and family obligation. In contrast, Filipino American families' socialization reflects the legacy of U.S. colonialism, and historical exposure to Western culture may lead more complex identity processes, including greater emphasis on assimilation (becoming American) and, in some cases, minimization of race and racism, though later generations increasingly engage in more explicit racial–ethnic and racial–civic socialization. These differences highlight the importance of disaggregated, historically grounded approaches to understanding racial–ethnic socialization in Asian American families. Because our subsample sizes were too small to permit separate tests across ethnic groups, future research should aim to investigate culturally specific messages and mechanisms within particular Asian ethnic groups using adequately powered samples. Such work may uncover intersectional patterns obscured when Asian Americans are treated as a single, aggregated category. Second, our sample consisted predominantly of Asian American families with higher socioeconomic status and educational attainment, limiting generalizability. The socioeconomic positioning of our sample may have influenced ERS practices and parent‐adolescent communication patterns. Future research should include more socioeconomically diverse samples to examine how socioeconomic status may influence ERS processes.

Third, the data used to identify profiles was cross‐sectional, preventing us from assessing the longer‐term implications of these profiles. A longitudinal design is needed to determine whether ERS profiles and their impact on youth development remain stable. Fourth, the smallest profile comprised only 5% of the sample (*n* = 24). Smaller profiles can be sensitive to sample‐specific characteristics, and the stability and generalizability of this profile structure should be examined in future studies. Fifth, the adolescent‐reported becoming American ERS subscale demonstrated borderline acceptable reliability (α = .64), and results involving this construct should be interpreted with caution. Sixth, we did not control for social desirability in youth or parents, which could have influenced the accuracy of ERS reports or our measure of academic performance, which relied on adolescent self‐reports. Finally, most parent participants were first‐generation immigrants. Future research should recruit second‐ or third‐generation Asian American families to examine the association between immigrant status and parent ERS practices.

## CONCLUSIONS

Our findings affirm and extend cultural‐ecological and integrative developmental theories by providing insights into how parent and adolescent experiences with ERS influence Asian American youth adjustment. Our study highlights the importance of considering both the content of ERS messages and the process of ERS transmission in understanding adolescent adjustment. Incorporating both parent and adolescent perspectives can provide a more comprehensive understanding of these family socialization processes. Profiles characterized by frequent positive ERS, infrequent negative ERS, and parent‐adolescent agreement on the frequency of ERS practices were associated with the most adaptive adolescent outcomes. The observed differences in outcomes across profiles reinforce the importance of clear parental communication that emphasizes positive ERS practices to facilitate shared understanding of race and ethnicity.

These findings can inform culturally responsive parenting interventions, assessments, and mental health programs for schools and families. Programs should provide guidance on not only the types of ERS messages to communicate, but also how parents may deliver clear, consistent, and developmentally appropriate ERS messages. Creating opportunities for open dialogue between parents and adolescents about their ethnic–racial experiences and perspectives may help improve communication and reduce discrepant perceptions in the family. Families characterized by higher levels of negative ERS and larger discrepancies between parent and youth perceptions of ERS may also be identified for interventions aimed at improving parent‐youth communication and youth development. Interventions for families resembling the Parent Low Youth Moderate Negative profile should consider targeting positive parent‐adolescent dialogue regarding race/ethnicity, as well as positive activities related to families' ethnic heritage culture. Practitioners can facilitate structured conversations between parents and their adolescents about race, ethnicity, and ethnic–racial discrimination and encourage continuity of these conversations at home. These conversations may include dialogue about what parents and adolescents believe it means to be a member of their ethnic–racial group and why their ethnic–racial identity is important. Furthermore, these conversations should include discussions about potential ethnic–racial discrimination and specific skills to respond to discriminatory experiences, such as seeking support from trusted adults. Activities that promote ethnic identity formation can include parents and adolescents participating in cultural activities and holidays, cooking and eating cultural foods, and reading books related to or written by members of their ethnic–racial group. Moreover, discussions regarding the historical contributions and struggles of members of their ethnic–racial group, both in their country of origin and in the United States, can also help facilitate ethnic–racial identity development, cultural pride, and a sense of belonging as an American and as a member of their ethnic–racial group. Parents and adolescents can discuss how they believe these historical events have impacted life for members of their ethnic–racial group today. In addition, parents should encourage their adolescents to accept and appreciate other ethnic–racial groups and cultures, emphasizing that diversity is a strength to individuals and communities (Atkin et al., 2019; Hughes et al., 2006).

Finally, these findings underscore the importance of capturing multiple dimensions of ERS, offering ways to tailor interventions and assessments more suitably for youth with different developmental needs. Practitioners can assess parent‐adolescent discrepancies in ERS practices in clinical settings by using validated measures, such as the Asian American Parental Racial–Ethnic Socialization Scale (AAPRES; Juang et al., 2016). They can also ask parents and youth questions about ERS practices at home to assess their agreement. This will allow them to highlight perceived discrepancies, negative consequences associated with negative ERS messages, and coach parents and youth to have more open, bidirectional, and positive conversations about race and ethnicity. Because adolescents tend to have more exposure to multicultural issues and are more aware of racial dynamics (Patel et al., 2023), practitioners can also encourage youth to share their multicultural knowledge with parents.

## AUTHOR CONTRIBUTIONS

The authors of this paper made substantial contributions to this manuscript and have approved the final submitted version. The first author conceptualized the research aims and methodology, conducted data analysis, and interpreted results. The second and third authors guided the whole research process and contributed to manuscript revision. The fourth and fifth authors conducted literature reviews and contributed to manuscript revision.

## FUNDING INFORMATION

This study was funded by Russell Sage Foundation (Y16CC20229).

## CONFLICT OF INTEREST STATEMENT

The authors report that there are no competing interests to declare.

## ETHICS STATEMENT

This study was approved by the IRB at the University of Maryland (Approved March 3, 2020).

## PATIENT CONSENT STATEMENT

Parental informed consent was obtained prior to data collection.

## Supporting information


Figure S1.

## Data Availability

The data that support the findings of this study are available from the corresponding author upon reasonable request.
