# Supplementary material for: Profiles of Asian American parent‐ and adolescent‐reported ethnic‐racial socialization: A person‐centered analysis
Source: J Res Adolesc. 2026 Apr 7;36(2):e70176. doi: 10.1111/jora.70176 (PMC13058401; doi:10.1111/jora.70176)
Supplement: Supplementary file 1 — Figure S1. [file JORA-36-0-s001.docx]

Supplemental Material

**Figure S1**

*Patterns of Parent- and Adolescent-Reported ERS by Profile*

*
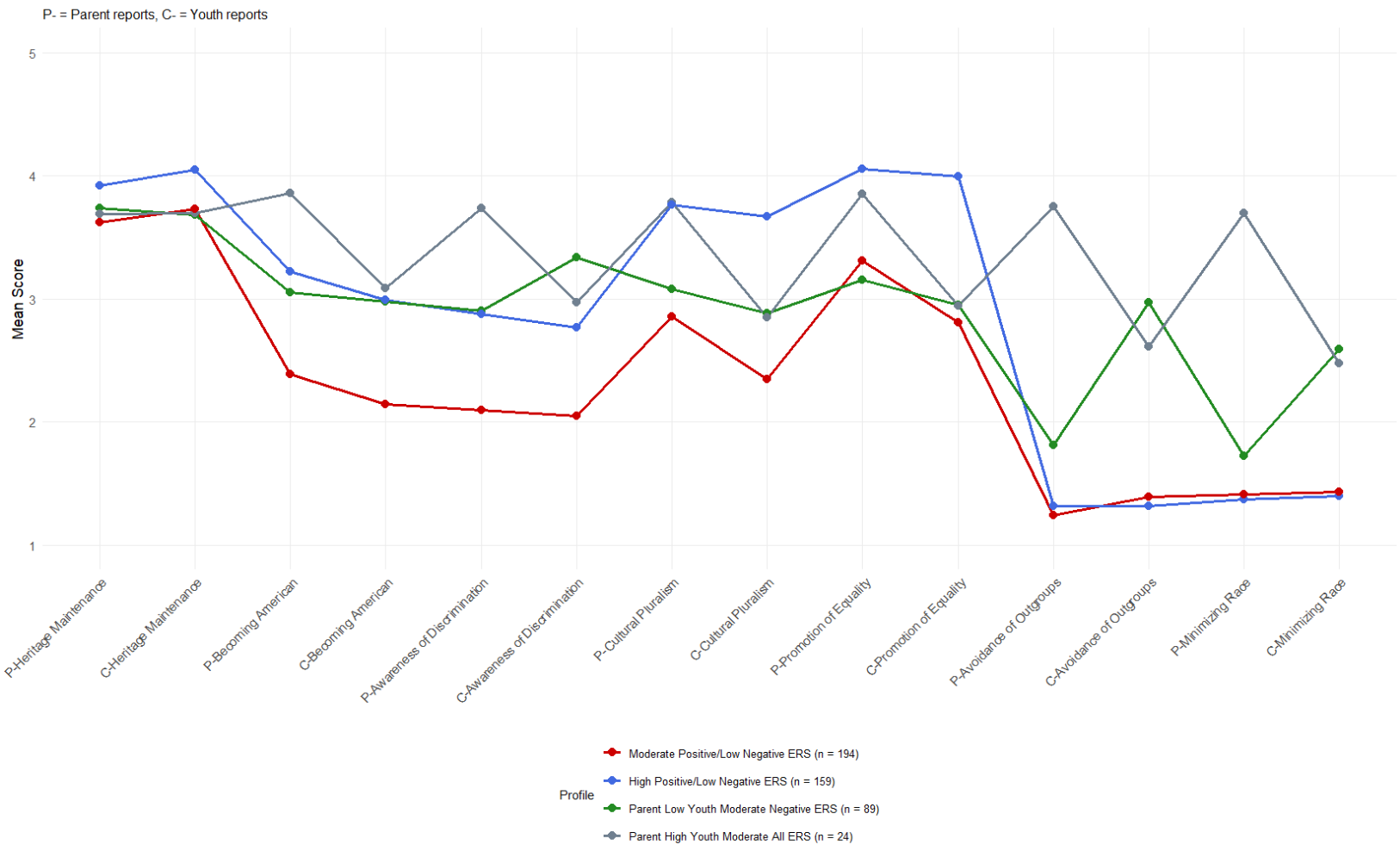
*

*Note.* Profiles are organized by color. ERS messages beginning with P- represent parent reports while messages beginning with C- represent child reports.
